# Exploring the interconnectedness of fatigue, depression, anxiety and potential risk and protective factors in cancer patients: a network approach

**DOI:** 10.1007/s10865-019-00084-7

**Published:** 2019-08-22

**Authors:** Melanie P. J. Schellekens, Marije D. J. Wolvers, Maya J. Schroevers, Tom I. Bootsma, Angélique O. J. Cramer, Marije L. van der Lee

**Affiliations:** 1 Scientific Research Department, Centre for Psycho-Oncology, Helen Dowling Institute, Professor Bronkhorstlaan 20, Postbus 80, 3720 AB Bilthoven, The Netherlands; 2 Department of Methodology and Statistics, School of Social and Behavioral Sciences, Tilburg University, Tilburg, The Netherlands; 3 Department of Health Psychology, University Medical Centre Groningen, University of Groningen, Groningen, The Netherlands; 4 Department of Cultural Studies, School of Humanities and Digital Sciences, Tilburg University, Tilburg, The Netherlands

**Keywords:** Cancer, Cancer-related fatigue, Anxiety, Depression, Acceptance, Helplessness, Coping, Network analysis

## Abstract

Researchers have extensively studied fatigue, depression and anxiety in cancer patients. Several risk and protective factors have been identified for these symptoms. As most studies address these constructs, independently from other symptoms and potential risk and protective factors, more insight into the complex relationships among these constructs is needed. This study used the multivariate network approach to gain a better understanding of how patients’ symptoms and risk and protective factors (i.e. physical symptoms, social withdrawal, illness cognitions, goal adjustment and partner support) are interconnected. We used cross-sectional data from a sample of cancer patients seeking psychological care (n = 342). Using network modelling, the relationships among symptoms of fatigue, depression and anxiety, and potential risk and protective factors were explored. Additionally, centrality (i.e. the number and strength of connections of a construct) and stability of the network were explored. Among risk factors, the relationship of helplessness and physical symptoms with fatigue stood out as they were stronger than most other connections in the network. Among protective factors, illness acceptance was most centrally embedded within the network, indicating it had more and stronger connections than most other variables in the network. The network identified key connections with risk factors (helplessness, physical symptoms) and a key protective factor (acceptance) at the group level. Longitudinal studies should explore these risk and protective factors in individual dynamic networks to further investigate their causal role and the extent to which such networks can inform us on what treatment would be most suitable for the individual cancer patient.

**Electronic supplementary material:**

The online version of this article (10.1007/s10865-019-00084-7) contains supplementary material, which is available to authorized users.

Receiving a cancer diagnosis has a major impact on patients’ lives. One of the most prevalent long-term side-effects of cancer and its treatment is fatigue. Patients who suffer from severe fatigue also often suffer from symptoms of depression and/or anxiety (Donovan et al., 2013; Hofman et al., 2007; Zhu et al., 2017). In addition, depression and anxiety often co-occur in cancer patients (Mitchell et al., 2011). Multiple studies have demonstrated high correlations among these three problem areas in cancer patients (Brown & Kroenke, 2009). Agasi-Idenburg et al. (2017) demonstrated that fatigue, depression and anxiety form a symptom cluster: a set of multiple co-occurring symptoms that are strongly interrelated (Miaskowski et al., 2017). Compared with a single symptom, the occurrence of symptom clusters appears to worsen patient outcomes as symptoms interact with one another. Longitudinal studies provide additional support for this clustering, such that when patients improve on either fatigue, depression or anxiety, they are likely to improve on the other problem areas as well (Zhu et al., 2017). These strong relationships between fatigue, depressive and anxiety symptoms is not surprising, given that “fatigue or loss of energy” is one of the three overlapping criteria (i.e. in addition to concentration problems and sleep problems) between major depression disorder (MDD) and generalized anxiety disorder (GAD), according to the Diagnostic Statistical Manual version 5 (DSM-5) (APA, 2013). In order to provide patients with the best possible care, we need a better understanding of how these symptoms are interconnected and what kind of risk and protective factors are related to these symptoms.

A relevant and well-known theory to understand patients’ adaptation to cancer and their functioning is the stress-coping model. The model predicts that a diagnosis of cancer can lead to symptoms of fatigue, depression, and/or anxiety, due to the appraisal of and patients’ coping with cancer and related stressful events (Maes et al., 1996). In line with the stress-coping model, several studies have successfully identified factors that put patients at risk or protect them from the development and persistence of fatigue, depression and anxiety. For example, several studies found evidence that indeed, patients’ appraisals or illness cognitions such as acceptance, helplessness and perceived benefits, are significantly related to patients’ reports of distress and fatigue (Evers et al., 2001; Hudson et al., 2018; Richardson et al., 2017; Westbrook et al., 2016). Moreover, the stress-coping model poses that the extent to which cancer patients are able to flexibly manage the goals that have become unattainable is a key factor in adaptively coping with cancer and related symptoms. Previous research in cancer patients has shown that being better able in such goal adjustment is related to less depressive and anxiety symptoms (Schroevers et al., 2011; Zhu et al., 2015). Finally, the stress-coping model states that additional stressful events in terms of somatic comorbidity as well as (a lack of) social support from the environment may impact adaptation. In cancer patients, it has been found that an overall poorer health and comorbidity are related to higher levels of fatigue, depression and anxiety (Husson et al., 2015; Zhu et al., 2017) and that social support (e.g. from the partner) is related to less psychological distress (Kamen et al., 2015; Kuijer et al., 2000).

While these studies have provided valuable insights into the risk and protective factors for the development and persistence of fatigue, depression and anxiety, current research findings are limited. With some notable exceptions (such as structural equation modeling), most studies address these constructs, independently from other symptoms and potential risk and protective factors. This most likely is not a reflection of reality. The notion that these symptoms are likely interrelated in more complex ways (e.g. fatigue → concentration problems → social withdrawal → worthlessness → depressed mood) and can have a reciprocal relationship (e.g. fatigue → depressed mood; depressed mood → fatigue) is ignored. To gain a more comprehensive understanding of the complex nature of how these symptoms and risk and protective factors are interconnected, it might be more suitable to take a network approach.

The network approach is a relatively new research area in clinical psychology and psychiatry, in which a mental disorder such as MDD is theorized to be the result of the causal interplay between symptoms in a network structure (Borsboom, 2017; Borsboom & Cramer, 2013; Cramer et al., 2010, 2016). A network structure consists of “nodes” representing the selected variables and “edges” representing the links that connect two nodes (e.g. regularized partial correlation coefficients). Thus, rather than viewing symptoms as manifestations of a common cause (i.e. you feel depressed because you have MDD), symptoms are conceptualized as elements of a complex dynamical system, in which symptoms are mutually interacting with one another (Borsboom & Cramer, 2013). That is, a certain symptom can activate other symptoms in the network (e.g. sleep problems can trigger fatigue and worthlessness, which in turn can trigger depressed mood and loss of enjoyment) and, in line with what has long been common knowledge among clinicians, symptoms can reinforce one another leading to symptom cycles. Consequently, such a network of strongly interconnected symptoms can fulfil the criteria of MDD (Borsboom, 2017). To date, the network approach has contributed to several advancements in psychopathology research [for a review of hallmark empirical insights, see Fried et al., 2017].

Recently, researchers have suggested to move beyond symptom measures and also include theoretically relevant nodes within the network to gain more insight into the nature of the relationships and core mechanisms involved in psychopathology (Borsboom, 2017; Fried & Cramer, 2017). By including other factors in addition to symptoms of psychopathology in the multivariate model, the network model provides a more integrative approach, making it ideally suited to study constructs that operate at the crossroads between psychology and health (Van der Lee & Schellekens, 2019). For example, it provides the opportunity to examine how bodily symptoms related to cancer are associated with symptoms of psychopathology. Also potential risk or protective factors related to appraisals of cancer (e.g. feeling helpless, acceptance of illness) and social support, can be added to the network model, allowing us to explore patients’ adjustment to cancer in its full complexity.

In order to study which symptoms activate or trigger one another, dynamic networks are needed, which requires intensive longitudinal study designs. However, network modeling can also be employed in cross-sectional designs to study the co-occurrence of certain concepts, offering insight into patterns of symptoms, risk and protective factors across individuals. Moreover, cross-sectional networks provide a valuable first step in designing longitudinal studies as they can offer insight into what concepts and what connections are of importance at the group level. The aim of the present study is to contribute to revealing the complex nature of the cross-sectional relationships among patients’ symptoms (i.e. fatigue, depression and anxiety) and potential risk and protective factors (i.e. physical symptoms, social withdrawal, illness cognitions, goal adjustment and partner support), by analysing the multivariate network structure of these symptoms and factors at the group level.

## Methods

### Participants and procedure

We analysed data from an observational cohort study (Garssen et al., 2016), which evaluated psychological care for cancer patients. Patients who applied for psychological care at one of the seven mental health care institutes specialised in psycho-oncology care (IPSO) in the Netherlands were consecutively sampled. The full sample consisted of patients with cancer (*n *= 384) and their partners (*n *= 99). In the present study, we used baseline data from the patient sample only. Inclusion criteria were: (1) diagnosed with cancer and seeking psychological help at one of the seven IPSO, (2) ≥ 18 years of age and (3) sufficient understanding of Dutch language. Patients who agreed to participate signed the informed consent form and were assessed before initiating psychological care. The study was approved by the Ethical Board of the Helen Dowling Institute. More detailed information on the study is described elsewhere (Garssen et al., 2016; Zhu et al., 2017).

### Measures

Socio-demographic (age, gender, relationship status, children at home, educational level, job status) and clinical characteristics (time since diagnosis, cancer type, metastasis, medical treatment and current treatment) were obtained through a self-report questionnaire.

#### Symptom measures

For an overview of the used questionnaires and selected nodes, see Online Supplementary Table 1. Symptoms were selected based on clinical relevance, assuring that the items did not overlap in content. That is, we selected eight symptom measures from the available dataset that matched a subset of the DSM-5 criteria of MDD and GAD (APA, 2013): fatigue (FATIG), depressed mood (DEPRE), loss of enjoyment (ENJOY), anxiety (ANXIE), sleep problems (SLEEP), concentration problems (CONCE), worthlessness (WORTH) and appetite loss (APPET). If there were more items representing a DSM criterion we averaged those items into one score. The following criteria of MDD or GAD were not assessed in the available dataset: suicidality, difficulty controlling anxiety, restlessness, irritability and muscle tension. Note that in the selection of symptoms and risk and protective factors we were limited by the size of the available sample. We ensured that at least the number of estimated parameters in the network did not exceed the number of cases (Epskamp et al., 2017).

To assess the MDD/GAD criteria of *Fatigue*, we used all of the 8-item Fatigue Severity subscale of the Dutch Checklist Individual Strength (CIS-FS) (Vercoulen et al., 1994). A sample item is “I feel tired”. For each item, participants indicated the extent to which it was true during the past 2 weeks. Response categories ranged from 1 (yes, that is true) to 7 (no, that is not true). The CIS has been validated and showed good psychometric properties in chronic fatigue syndrome patients and the general population (Vercoulen et al., 1994; Worm-Smeitink et al., 2017). Internal consistency was good in the present sample (α = .92).

Depressive and anxiety symptoms were measured with 8 items of the 16-item version of the Center for Epidemiologic Studies Depression Scale (CES-D). The Dutch version of the CES-D has been validated in cancer patients and healthy controls and shows good psychometric properties (Schroevers et al., 2000). To assess *Depressed Mood* the following items were averaged: “I felt I could not shake of the blues”, “I felt depressed” and “I felt sad” (α = .85 in the present sample). Note that the CES-D does *not* use as skip structure when the basic criteria of MDD (depressed mood and loss of enjoyment) are not met. To measure *Sleep Problems* the item “My sleep was restless” was used. To measure *Concentration Problems* the item “I had trouble keeping my mind on what I was doing” was used. To assess feelings of *Worthlessness* the item “I thought my life had been a failure” was used. To measure *Appetite Loss* the item “I did not feel like eating; my appetite was poor” was used. One item of the CES-D was used to measure *Anxiety*: “I felt fearful”. We choose an item of the CES-D rather than the total scale of the 6-item State Anxiety Inventory (SAI) (Korfage et al., 2006) because the SAI refers to anxious feelings from a different timeframe than the other symptom measures (i.e. the present moment rather than the past week/weeks). Participants scored how often they experienced a symptom in the past week, ranging from 0 (rarely or none of the time) to 3 (most or all of the time).

To assess the MDD criteria *Loss of Enjoyment* (De Bruin et al., 1996) we used the Wellbeing subscale of the Dutch Health and Disease Inventory (HDI). The HDI has shown good psychometric properties (De Bruin et al., 1996). We selected four (out of 13) items that focus on enjoyment: “I have lots of plans”, “I enjoy the things I do”, “I have pleasant things to look forward to”, “I enjoy my life” (α = .82 in present sample). Scores were recoded such that a higher score portrayed less enjoyment. Each item was rated to how often it was true in the past four weeks, ranging from 1 (never) to 6 (always).

#### Measures of risk and protective factors

Based on the stress-coping model and previous findings (e.g. Evers et al., 2001; Kamen et al., 2015; Zhu et al., 2015, 2017), risk and protective factors were chosen from the dataset. This led to the following selection of risk factors (physical symptoms (PHYSI), social withdrawal (SOCIAL), and helplessness (HELPL)) and protective factors (acceptance of illness (ACCEPT), perceived benefits of illness (BENEF), disengagement of unattainable goals (DISENG) and reengagement of new goals (REENG)) for the network analysis. Additional analyses also included three partner support factors: active engagement (P-ACTI), protective buffering (P-BUFF) and overprotection (P-OVER).

*Physical Symptoms* were measured with the Rotterdam Symptom Checklist (RSCL) (de Haes et al., 1990). Previous validation studies in cancer patients showed good psychometric properties (de Haes et al., 1990). We selected the following nine (out of twelve) items: “nausea”, “headache”, “stomach ache”, “shivering”, “tingling”, “shortness of breath”, “dizziness”, “diarrhoea” and “constipation” (α = .74 in the present sample). Because of the limited number of variables that can be included in the network, we averaged these symptoms into one variable. Three items were not selected because the symptoms (i.e. appetite loss, insomnia, concentration problems) were already covered by the CES-D. Patients indicated to what extent they were bothered by the symptom in the past week from 0 (not at all) to 4 (very much).

*Social Withdrawal* was assessed with all items of the 8-item subscale Social Roles of the Dutch Groningen Social Behaviour Questionnaire (GSBQ) (De Jong & Van der Lubbe, 2001; Van der Lubbe, 1995). A sample item is “I contacted my friends and close acquaintances less than usual”. Participants indicated to what extent they agreed with the statement in the past 4 weeks, ranging from 1 (strongly disagree) to 5 (strongly agree). The GSBQ has been validated in psychiatric patients, showing good psychometric properties (De Jong & Van der Lubbe, 2001; Van der Lubbe 1995). In the present study internal consistency was good (α = .91).

The illness cognitions *Helplessness, Acceptance of Illness,* and *Perceived Benefits of Illness* were assessed with all items of the corresponding subscales of the 18-item Dutch Illness Cognitions Questionnaire (ICQ). Helplessness is considered a way of emphasizing the negative meaning of the disease, Acceptance as a way to diminish the aversive meaning, and Perceived Benefits as a way of adding positive meaning to the disease. A sample item of each subscale is “My illness makes me feel useless at times”, “I have learned to live with my illness”, “Dealing with my illness has made me a stronger person”, respectively. Each item is answered to the extent to which one agrees with the item, ranging from 1 (not at all) to 4 (completely). The ICQ was validated in a sample of patients with rheumatoid arthritis and multiple sclerosis and showed to be valid and reliable. In the present study the subscales Helplessness (α = .84), Acceptance (α = .89) and Perceived Benefits (α = .86) showed good internal consistency.

*Goal Disengagement* and *Goal Reengagement* were assessed with all items of the corresponding subscales of the 10-item Goal Adjustment Scale (GAS) (Wrosch et al., 2003). The 4-item subscale Goal Disengagement measures the ease with which participants can give up efforts and commitment towards unattainable goals. A sample item is ‘When I could no longer pursue this goal, it was easy for me to reduce effort towards the goal’. The 6-item subscale Goal Reengagement measures the extent to which patients reengage into new attainable goals when confronting unattainable goals. A sample item is ‘When I could no longer pursue this goal, I put effort towards other meaningful goals’. Participants rated to what extent they agreed with each item, ranging from 1 (strongly disagree) to 5 (strongly agree). The GAS has been validated in the general population as well as in cancer patients, showing good psychometric properties (Schroevers et al., 2011; Wrosch et al., 2003). Internal consistency of the subscales Goal Disengagement (α = .82) and Goal Reengagement (α = .87) was good in the present study.

Partner support in terms of *Active Engagement*, *Protective Buffering* and *Overprotection* was assessed with all items of the corresponding subscales of the 19-item Ways of Giving Support (WGS) questionnaire (Buunk et al., 1996). The 5-item Active Engagement subscale assesses constructive problem-solving methods of giving support, such as involving the patient in discussions. The 8-item Protective Buffering subscale measures behaviour like hiding concerns and yielding to the patient in order to avoid disagreements. The 6-item Overprotection subscale measures an underestimation of the patients’ capabilities, resulting in unnecessary help or attempts to restrict activities. Sample item of the subscales are “My partner asks me how I feel”, “My partner tries to hide his or her worries about me”, “When it comes down to it, my partner seems to think that I don’t know what’s right for me”, respectively. Patients were asked to rate to what extent their partner adopted these ways of giving support, ranging from 1 (never) to 5 (very often). Psychometric properties of the WGS seem adequate (Buunk et al., 1996; Kuijer et al., 2000). Internal consistency of the subscales Active Engagement (α = .73), Protective Buffering (α = .58) and Overprotection (α = .72) were adequate to good in the present sample.

### Statistical analysis

The data analysis plan was pre-registered online at Open Science Framework, before we commenced data analysis (https://osf.io/5r3pn/). We estimated two network models: (1) a network with symptoms, risk and protective factors, based on complete cases (i.e. main sample) and (2) a network with partner support in addition to the symptoms and factors explored in the first network, based on complete cases from patients with a partner (i.e. relationship sample). As the data were not multivariate normally distributed, a nonparanormal transformation was applied to relax the normality assumption prior to estimating the networks (Liu et al., 2009).

#### Network estimation

The regularized partial correlation coefficients networks were estimated using the R package qgraph (Epskamp et al., 2012; Epskamp & Fried, 2016; R Core Team, 2016). In the network, “nodes” represent the selected variables while “edges” (links connecting two nodes) represent the regularized partial correlation coefficients. In regularized partial correlation networks the association between two nodes is estimated while controlling for all other nodes. As such, edges in these networks can be interpreted as conditional (in)dependence relations: if an edge is absent between two nodes, this means that these two nodes are conditionally independent given all other nodes. If an edge is present between two nodes, this means that these two nodes are conditionally dependent given all other nodes in the network.

To control for spurious connections that may result from sampling error (Costantini et al., 2015), we applied the least absolute shrinkage and selection operator (LASSO) (Tibshirani, 1996). The LASSO is a regularization method, leading (small) edge estimates to shrink to exactly zero. Consequently, the LASSO returns a sparse and more interpretable network model. To control the degree to which regularization is applied the LASSO utilizes a tuning parameter, which can be selected by minimizing the Extended Bayesian Information Criteria (EBIC). The qgraph package in R (Epskamp et al., 2012) combines the graphical LASSO (glasso, a well-established and fast algorithm for estimating LASSO regularization) (Friedman et al., 2008) with EBIC model selection (using the default value of hyperparameter γ = 0.5) to estimate a regularized partial correlation network.

The R package bootnet (Epskamp et al., 2017) was used to explore the stability of the network parameters. To estimate the accuracy of edge weights, bootnet estimates 95% bootstrapped confidence intervals around each edge in the network. The package also provides significance tests to examine whether certain edges are stronger than other edges, based on the bootstrapping results. Only the edges that prove to be significantly stronger than most other edges will be interpreted as such.

#### Node centrality

To examine the importance of each node in the network we estimated three indices of node centrality (Opsahl et al., 2010): node strength, betweenness and closeness. In a weighted network, node strength refers to the number and strength of the direct connections of a node. Betweenness is a measure of how often a node lies on the shortest path between every combination of two other nodes, indicating to what extent the node facilitates the flow of information through the network. Closeness measures the average distance from a node to all other nodes in the network, representing how fast a node can be reached from the other nodes in the network. To estimate the stability of node centrality, the R package bootnet provides the central stability coefficient (CS-coefficient), which is estimated based on a subsetting bootstrapping procedure. The CS-coefficient represents the proportion of participants that can be dropped from the analysis, such that the correlation between the original centrality indices and the subset centrality indices is at least 0.7 with 95% probability (Costenbader & Valente, 2003). Only the centrality measures with a CS-coefficient ≥ 0.25 will be interpreted.

## Results

### Study sample

Of the 384 patients who filled out the baseline questionnaire, 342 (89%) were complete cases and were therefore included in the analysis of the main sample. The complete cases did not differ from the patients with missing data regarding sociodemographic, clinical and psychological characteristics (*p* > .05), except for hormone treatment. Patients included in the analysis were more often treated with hormone treatment than excluded patients (respectively 26.2% vs. 9.5%, *p* = .018). Patients in the main sample, of whom 77.2% were female, had a mean age of 51.35 years (SD = 10.62). Patients were mainly diagnosed with breast cancer (45.6%). In 36.8% of cases the cancer had metastasized and 46.5% received current treatment for their cancer. Of all patients, 58.5% was considered severely fatigued (CIS-SF ≥ 35), 68.1% was depressed (total CES-D ≥ 10) and 56.1% was anxious (SAI ≥ 14) (Korfage et al., 2006). See Table 1 for baseline characteristics. Table 2 presents the mean scores of the nodes used in the networks.

**Table 1:** Demographic and clinical characteristics of 342 cancer patients

	n (%)
Age [M (SD)]	51.35 (10.62)
Female gender	264 (77.2)
In a relationship^a^	271 (79.2)
Educational level^bc^	
Low	38 (11.1)
Intermediate	129 (37.7)
High	173 (50.6)
Paid job	203 (59.4)
Absenteeism due to cancer past month^a^	164/203 (80.8)
Months since diagnosis [M (SD)]^d^	37.46 (64.30)
Cancer type^e^	
Breast	156 (45.6)
Digestive system	37 (10.8)
Lung	22 (6.4)
Hematologic	44 (12.9)
Head and neck	26 (7.6)
Gynaecological	31 (9.1)
Other types	62 (18.2)
Cancer recurrence	53 (15.5)
Cancer metastases^b^	126 (36.8)
Medical treatment^fg^	
Surgery	255 (74.6)
Chemotherapy	207 (60.5)
Radiotherapy	166 (48.5)
Hormone treatment	89 (26.0)
Immunotherapy	16 (4.7)
Bone marrow transplant	8 (2.3)
Other treatment	52 (15.2)
Current treatment^h^	159 (46.5)

^a^1 missing

^b^2 missing

^c^Low = primary and lower secondary education, intermediate = upper secondary education, high = higher vocational training/university

^d^100 missing

^e^Percentages do not add up to 100 because 33 patients had multiple types of cancer

^f^4 missing

^g^Percentages do not add up to 100 because patients followed multiple treatments

^h^34 missing

**Table 2:** Labels and mean scores of the selected nodes

Node (range)	M (SD)
Fatigue (7–56)	36.76 (12.25)
Depressed mood (0–9)	3.15 (2.43)
Loss of enjoyment (4–24)	12.93 (4.14)
Anxiety (0–3)	1.03 (0.94)
Sleep problems (0–3)	1.44 (1.00)
Concentration problems (0–3)	1.41 (0.90)
Worthlessness (0–3)	0.58 (0.86)
Appetite loss (0–3)	0.42 (0.73)
Physical symptoms (0–36)	14.08 (4.06)
Social withdrawal (8–40)	20.37 (7.48)
Helplessness (6–24)	12.92 (3.95)
Acceptance of illness (6–24)	12.80 (3.72)
Perceived benefits of illness (6–24)	13.96 (4.27)
Disengagement of unattainable goals (4–20)	10.84 (3.05)
Reengagement of new goals (6–30)	20.56 (3.85)

Of the 342 complete cases in the first network, 71 were dropped because they had no partner and 3 were dropped because they had missing data on the partner support variables, leaving 268 (72%) complete cases to be included in the relationship sample. Patients included in the relationship sample did not differ from those excluded from the relationship sample (*p* > .05), except for having a job. Included patients more often had a paid job than excluded patients (respectively 62.3% vs. 48.6%, *p* = .034).

### Relationship among symptoms, risk and protective factors

**Fig. 1:**
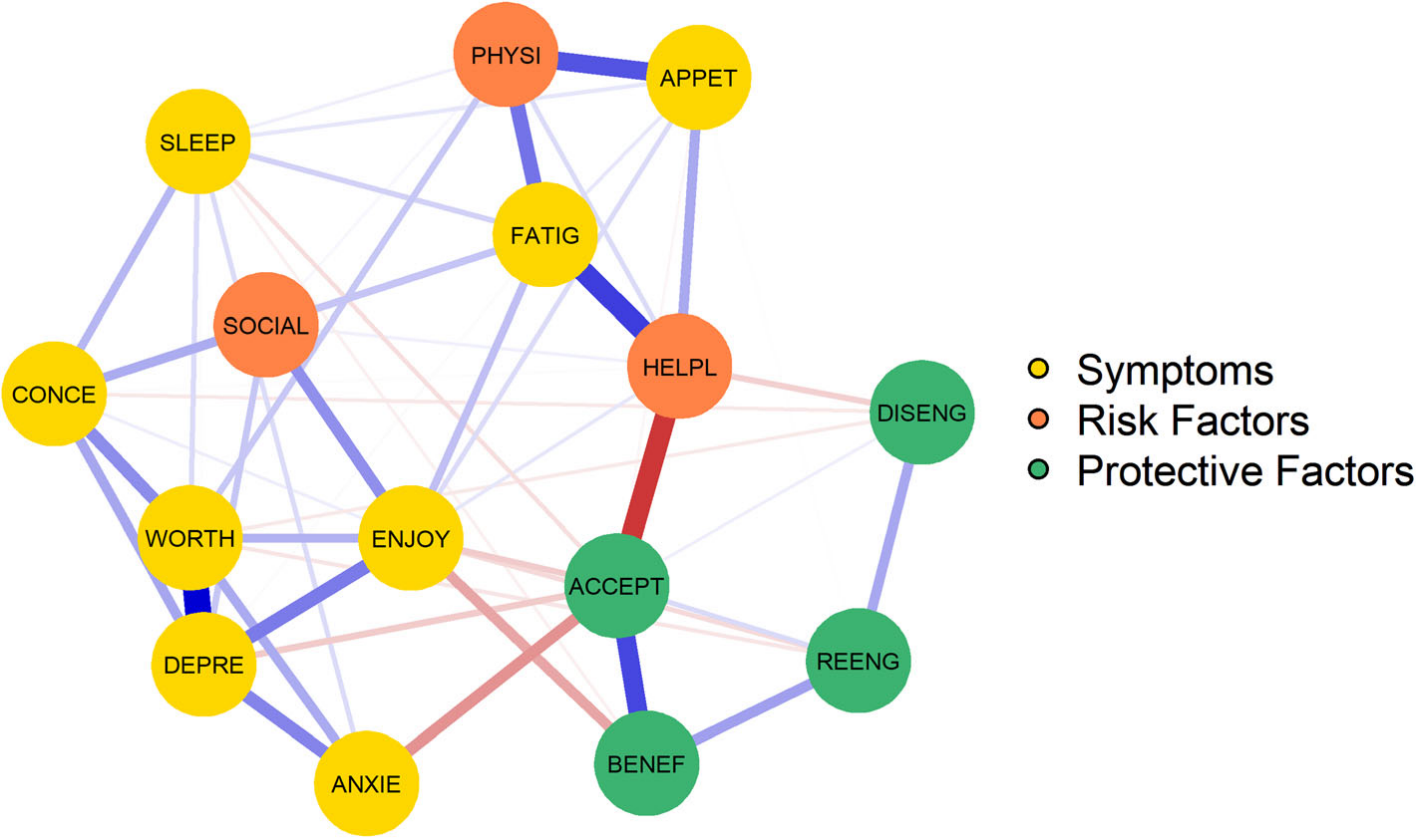
The network structure of symptoms and risk and protective factors of 342 cancer patients. The stronger a connection between two nodes, the thicker and more saturated the edge. Positive and negative connections are denoted by blue and red edges, respectively. FATIG = fatigue, DEPRE = depressed mood, ENJOY = loss of enjoyment, ANXIE = anxiety; SLEEP = sleep problems, CONCE = concentration problems, WORTH = worthlessness, APPET = appetite loss, PHYSI = physical symptoms, SOCIAL = social withdrawal, HELPL = helplessness, ACCEPT = acceptance of illness, BENEF = perceived benefits of illness; DISENG = disengagement of unattainable goals; REENG = reengagement of new goals

The regularized partial correlation network is presented in Fig. 1. Based on the 95% bootstrapped CI, the edge weights appeared rather stable (Online Supplementary Figure 2). Significance tests of edge weight differences (Online Supplementary Figure 3) indicated that the seven thickest and most saturated edges were significantly stronger than most other edges (i.e. 33–51) in the network: *Depressed Mood*—*Worthlessness, Depressed Mood*—*Loss of Enjoyment, Acceptance*—*Perceived Benefits, Acceptance*—*Helplessness, Fatigue*—*Helplessness, Fatigue*—*Physical Symptoms, Appetite loss*—*Physical Symptoms*. Many of the remaining edges were not reliably different from other edges, that is ten edges were significantly stronger than a few other edges (i.e. 1–23) and 39 edges were not stronger than any other edge.

### Network centrality

**Fig. 2:**
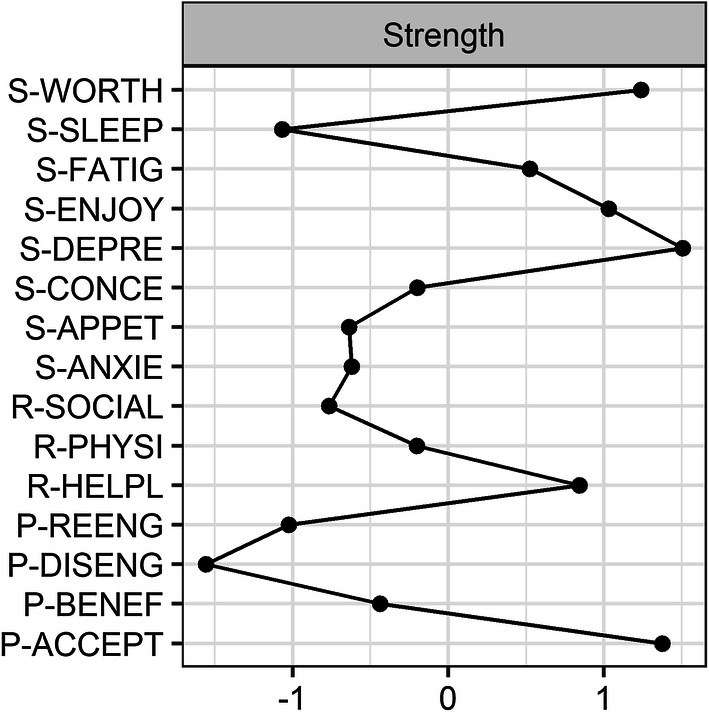
Strength centrality of each node in the network. Node strength refers to the number and strength of the direct connections of a node

The CS-coefficients for strength, closeness, and betweenness were 0.59, 0.44 and 0.21, respectively (Online Supplementary Figure 4), indicating that strength was the most reliable centrality index (see Fig. 2, for the other centrality indices see Online Supplementary Figure 5). Significance tests of differences in strength (Online Supplementary Figure 6) indicated that *Depressed Mood*, *Worthlessness*, *Loss of Enjoyment* and *Acceptance* were more central than most other nodes, that is these nodes had more and stronger connections with both symptoms and risk and protective factors than most other nodes in the network. *Sleep problems*, *Goal Reengagement*, *Goal Disengagement* and *Social Withdrawal* were the least central nodes in the network.

### Relationship among symptoms, risk and protective factors, including partner support

The regularized partial correlation network of the relationship sample was similar to the network of the main sample. The nodes on partner support (*Active Engagement, Protective Buffering, Overprotection*) were peripheral in the network. See Online Supplementary Material 7 for the results.

## Discussion

The present study applied a network approach to examine the interconnectedness among risk and protective factors on the one hand and symptoms of fatigue, depression and anxiety on the other hand, in cancer patients seeking psychological care. The network revealed that the symptoms depressed mood, loss of enjoyment and worthlessness were central nodes in the network, meaning that these symptoms had more and stronger connections than most other nodes in the network. Regarding risk factors, the relationships of helplessness and physical symptoms with fatigue were among the strongest connections in the network. The node physical symptoms was also strongly associated with appetite loss. Among the protective factors, acceptance of illness was centrally embedded in the network. Thus, while taking into account several risk and protective factors, acceptance of illness was most strongly connected to both symptoms as well as other risk and protective factors. The symptom sleep problems and the factors related to goal adjustment, social withdrawal and partner support appeared peripheral in the network and were less strongly associated with other nodes.

The present finding that depressed mood and loss of enjoyment are centrally embedded in the network correspond with the diagnostic criteria of MDD, which require that either depressed mood or loss of interest/pleasure are present. In other studies depressed mood and loss of interest/pleasure have also shown high centrality in both healthy controls as well as psychiatric patients (Boschloo et al., 2016; Fried et al., 2016). Regarding the connection between depressed mood, anxiety and fatigue, we found that anxiety and depressed mood are strongly related to one another but not to fatigue. This is in line with a recent study in a mixed sample of psychiatric patients and healthy controls, who also found that anxiety and depressed mood are mainly connected to fatigue via loss of enjoyment (Bekhuis et al., 2016). Other studies with samples of depressed patients, however, found different patterns regarding the connection between fatigue and depression (Beard et al., 2016; McWilliams et al., 2017).

Overall, the symptoms of fatigue, anxiety and depression appear to be strongly interconnected. Past research efforts may have overlooked the implications of conceptualizing psychiatric disorders as mutually interacting symptoms in cancer patients. While the literature has concluded that patients with cancer are vulnerable to develop psychiatric disorders (Mitchell et al., 2011), such as MDD, the network perspective might offer us an understanding into why these patients are vulnerable: cancer might lead to certain symptoms of MDD, which, in turn, can trigger other symptoms and eventually develop into a network of symptoms that correspond with the diagnostic criteria of MDD (Guloksuz et al., 2017; Van der Lee & Schellekens, 2019). This hypothesis is reflected in the present findings, showing that physical symptoms are strongly connected with fatigue and appetite problems, which in turn are related to other depressive symptoms. Note, however, that the cross-sectional design prevents us from drawing conclusions on any potential causal nature of these relationships.

Among the risk factors, the relationship between helplessness and fatigue stood out. This is in line with previous qualitative research showing how cancer related fatigue (CRF) is often experienced as uncontrollable, unpredictable and unchangeable, making patients feel helpless and distressed (Hofman et al., 2007; Scott et al., 2011). Among protective factors, acceptance of illness was centrally embedded in the network. This confirms previous bivariate research showing that coming to terms with one’s illness and its consequences is associated with a variety of physical and psychological health indicators, such as decreased anxiety and depression, adjustment to disease, and improved quality of life (Chabowski et al., 2017; Evers et al., 2001; Li & Moore, 1998; Peters et al., 2014). Furthermore, interventions targeting acceptance, such as Mindfulness-Based Cognitive Therapy, are effective in reducing fatigue, depression and anxiety (Bruggeman-Everts et al., 2017; Compen et al., 2018; Piet et al., 2012; Schellekens et al., 2017). Contrary to our expectations, the partner support factors were not strongly connected to symptoms and appeared peripheral in the network. This indicates that partner support is associated with patients’ symptoms and functioning but does not seem to play a central role in fatigue, depression and anxiety. A possible explanation could be that all other nodes in the network reflect patients’ thoughts, feelings and behaviour while partner support reflects patients’ perception of their partners’ behaviour. Future studies could further explore the role of the partner by studying other aspects of the relationship (e.g. relationship satisfaction, intimacy, communication) in a network perspective on cancer patients’ functioning.

A key hypothesis of the network approach is that by identifying and subsequently intervening on key nodes or connections in the network it should be possible to modify the behaviour of the network. That is, intervening on certain aspects of a network structure may serve to make the system, or network, return to a healthier state (e.g. no case of CRF or MDD). It would seem likely that for treatment to be successful, therapists could target (central) nodes in the network or specific relationships between nodes. However, note that in order to make statements regarding the suitability of treatment we need to study the dynamic networks of individual patients rather than group-level networks (Borsboom, 2017; Borsboom & Cramer, 2013). Future studies exploring individual dynamic networks can provide information on which symptoms and which risk and protective factors play a key role in the network for a specific individual, informing us which nodes and connections could and should be intervened upon, and consequently, what kind of treatment would be best suited for him or her. Given the present findings, acceptance and helplessness would be prime candidates to explore in these future studies.

The present study is the first to provide a network perspective on how risk and protective factors are related to key symptoms in cancer patients seeking psychological care. Moreover, the used questionnaires were selected based on their importance in clinical practice. We were able to analyse the stability of the estimated networks and identify differences between how central and how strongly connected certain symptoms and risk and protective factors were (Epskamp et al., 2017). Besides these strengths, some limitations should be taken into account. The study sample consisted of distressed cancer patients seeking psychological care. Consequently, findings cannot be generalized to distressed patients who are not seeking help. In addition, while the network models estimate how symptoms and factors are interrelated at a certain moment in time, the associations between such variables may be different when observed over multiple time points. Importantly, the cross-sectional between-subjects design allows for estimating conditional dependence relations, which are consistent with causal hypotheses about these relations but not sufficient to base causality on. In addition, as is the case for any cross-sectional model—whether it be networks or, say, factor models—cross-sectional results do not readily generalize to individuals (Bos & Wanders, 2016). Therefore, future studies could employ time-series designs, such as experience sampling (for an overview of how this method is applied in cancer research, see Kampshoff et al., 2019), in order to estimate dynamic networks for individuals in which an edge denotes a predictive relation (e.g. more fatigue in the morning predicts higher depressed mood in the afternoon).

## Conclusions

In conclusion, the network of symptoms and risk and protective factors identified depressed mood, worthlessness and loss of enjoyment as the most strongly connected symptoms in cancer patients seeking psychological care. Regarding the risk and protective factors, the relationships of helplessness and physical symptoms with fatigue were amongst the strongest connections in the network. The extent to which patients accept the cancer appeared highly embedded in the network. Longitudinal studies should explore these constructs in individual dynamic networks to further investigate their causal role and the extent to which such networks can inform us on what treatment would be most suitable for the individual cancer patient.

## Electronic supplementary material

Below is the link to the electronic supplementary material.

Supplementary material 1 (DOCX 3256 kb)
