# Supplementary material for: Exploring the interconnectedness of fatigue, depression, anxiety and potential risk and protective factors in cancer patients: a network approach
Source: J Behav Med. 2019 Aug 22;43(4):553–63. doi: 10.1007/s10865-019-00084-7 (PMC7366596; doi:10.1007/s10865-019-00084-7)
Supplement: Supplementary file 1 — Supplementary material 1 (DOCX 3256 kb) [file 10865_2019_84_MOESM1_ESM.docx]

**Online Supplementary Table 1.**

| **Questionnaires** | **Likert** | **Period** | **Corresponding nodes** | **α*** |
| --- | --- | --- | --- | --- |
| **Symptom measures** |  |  |  |  |
| 8-item Fatigue Severity subscale, Checklist Individual Strength (CIS-FS) | 7 | Past 2 weeks | - Fatigue: 8-item subscale Fatigue Severity | .92 |
| 16-item Center for Epidemiologic Studies Depression Scale (CES-D) | 4 | Past week | - Depressed mood: 3 items (blues, depressed, sad)  - Anxiety: 1 item (fearful)  - Sleep problems: 1 item (restless sleep)  - Concentration problems: 1 item (trouble keeping mind on task)  - Worthlessness: 1 item (life as failure)  - Appetite loss: 1 item (poor appetite) | .85 |
| 13-item Joy-in-Life subscale, Health and Disease Inventory (HDI) | 6 | Past 4 weeks | - Loss of enjoyment (recoded from Enjoying life): 4 items (full of plans, enjoying things, looking forward to pleasant things, enjoying life) | .82 |
| **Risk and Protective factors** |  |  |  |  |
| 12 items of the 23-item Physical Symptom Distress subscale, Rotterdam Symptom Checklist (RSCL) | 4 | Past week | - Physical symptoms: 9 items (nausea, headache, stomach ache, shivering, tingling, shortness of breath, dizziness, diarrhea, constipation) | .74 |
| 8-item Social Roles subscale, Groningen Social Behavior Questionnaire (GSBQ) | 5 | Past 4 weeks | - Social withdrawal: 8-item subscale Social Roles | .91 |
| 18-item Illness Cognitions Questionnaire (ICQ) | 4 | In general | - Helplessness: 6-item subscale Helplessness  - Perceived benefits of illness: 6-item subscale Perceived Benefits  - Acceptance of illness:6-item subscale Acceptance | .84  .89  .86 |
| 10-item goal adjustment Scale (GAS) | 5 | In general | - Goal disengagement: 4-item subscale Goal Disengagement  - Goal reengagement: 6-item subscale Goal Reengagement | .82  .87 |
| 19-item Ways of Giving Support (WGS) | 4 | In general | - Active engagement: 5-item subscale Active Engagement  - Protective buffering: 8-item subscale Protective Buffering  - Overprotection: 6-item subscale Overprotection | .73  .58  .72 |

***** Cronbach’s alpha in present sample.

**Online Supplementary Figure 2.** The 95% confidence intervals of the edges.


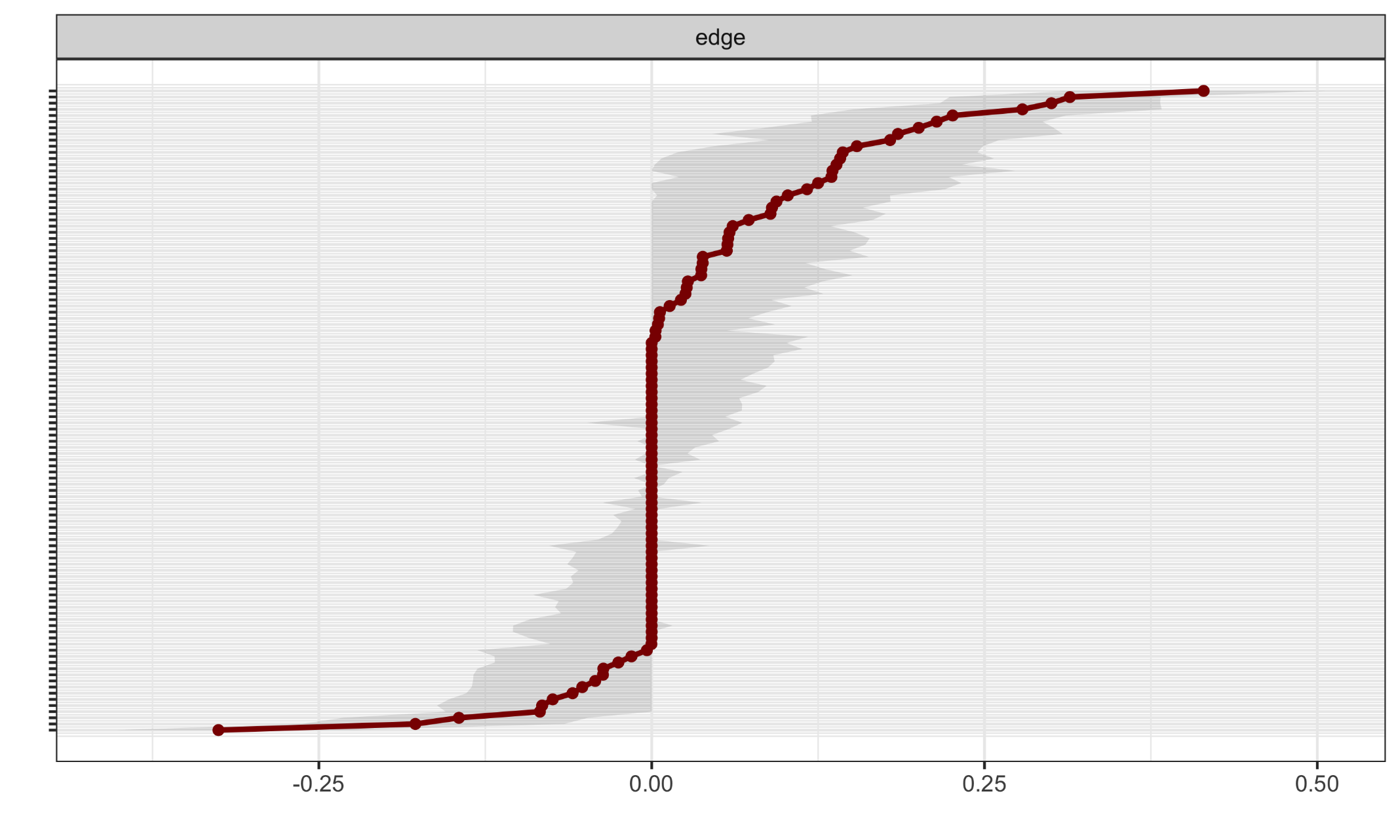

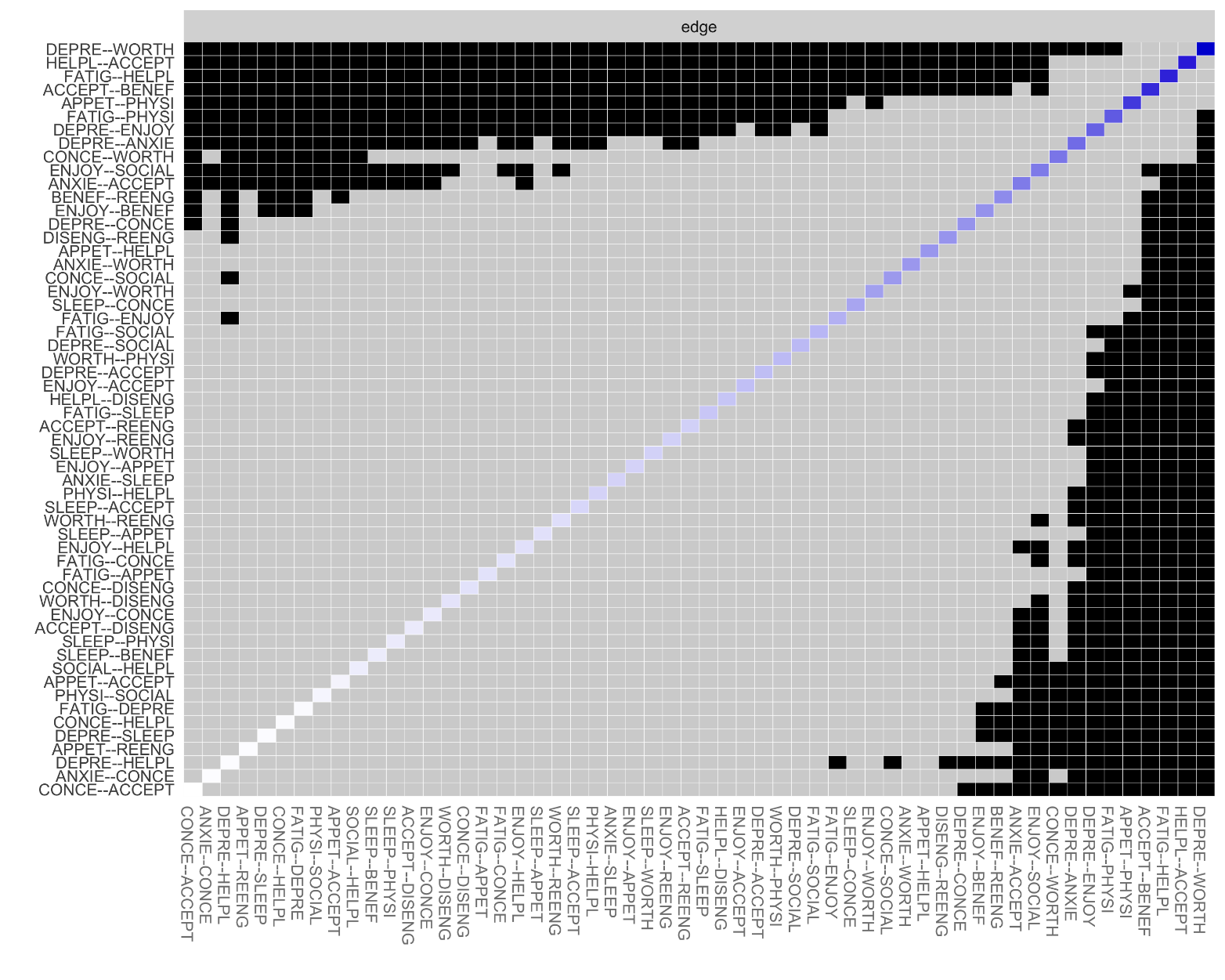
**Online Supplementary Figure 3.** Differences between edge weights.

Note. A black square depicts a significant difference between two edges. A grey square depicts a non-significant difference between two edges. Note that in order to compare the strength of the edges all positively coded variables (i.e. the protective factors) were negatively recoded.

**Online Supplementary Figure 4.** Stability of centrality indices.


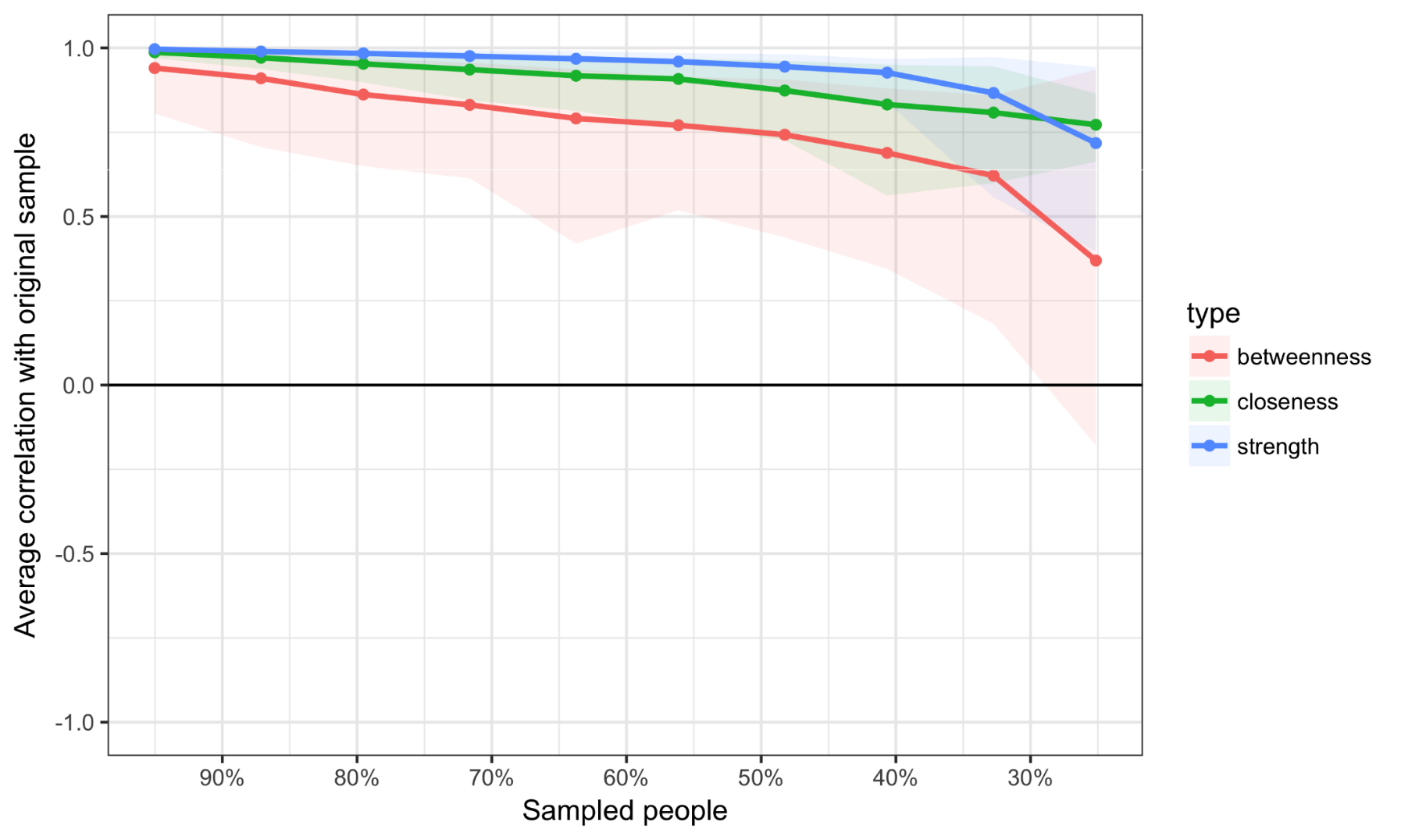


**Online Supplementary Figure 5.** Centrality of each node in the network.

**
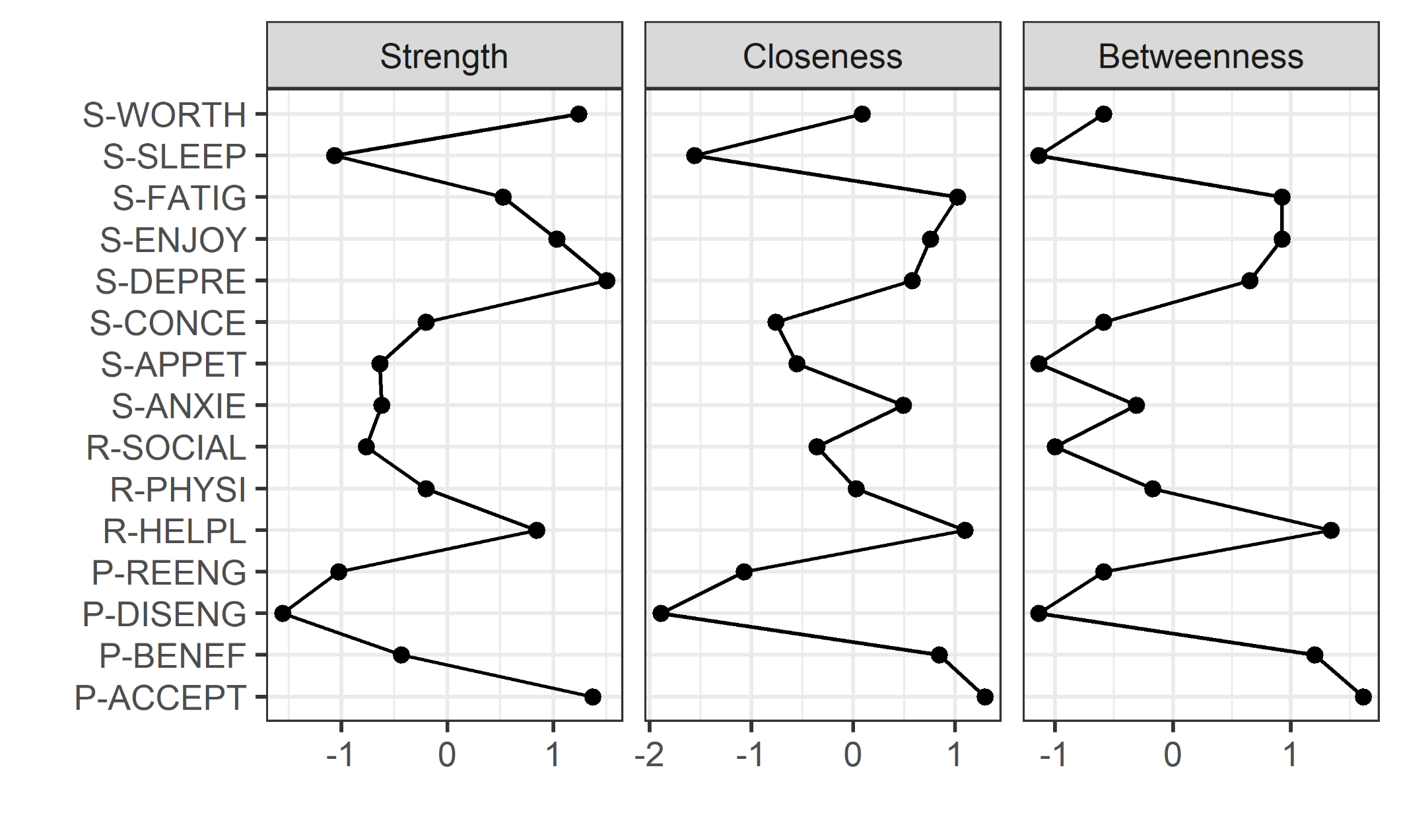
**

**Online Supplementary Figure 6.** Differences in strength centrality.


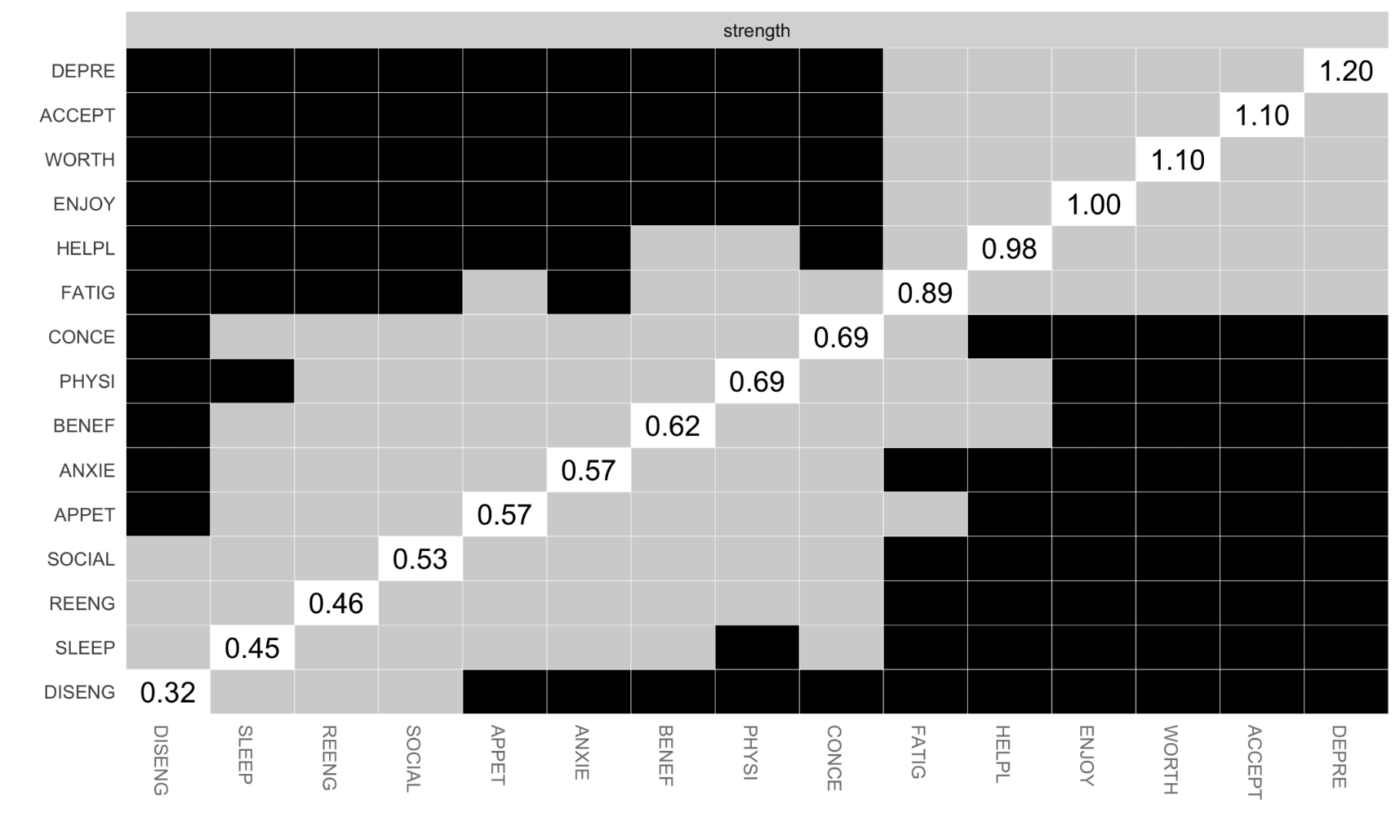


Note. A black square depicts a significant difference in strength centrality between two nodes. A grey square depicts a non-significant difference between two nodes.

**Online Supplementary Table 7.1** Demographic and clinical characteristics of patients in the partner sample.

|  | Partner sample (n=268)  n (%) |
| --- | --- |
| Age, M (SD) | 51.13 (10.23) |
| Female gender | 202 (75.4) |
| In a relationship ^a^ | 268 (100.0) |
| Educational level ^bc^ |  |
| Low | 29 (10.8) |
| Intermediate | 101 (37.7) |
| High | 136 (50.7) |
| Paid Job | 167 (62.4) |
| Absenteeism due to cancer past month ^a^ | 131/167 (78.9) |
| Months since diagnosis, M (SD) ^d^ | 39.65 (67.04) |
| Cancer type ^e^ |  |
| Breast | 122 (45.5) |
| Digestive system | 28 (10.4) |
| Lung | 17 (6.3) |
| Hematologic | 38 (14.2) |
| Head and neck | 21 (7.8) |
| Gynaecological | 22 (8.2) |
| Other types | 49 (18.3) |
| Cancer recurrence | 38 (14.2) |
| Cancer metastases ^b^ | 95 (35.4) |
| Medical treatment ^fg^ |  |
| Surgery | 197 (73.5) |
| Chemotherapy | 169 (63.1) |
| Radiotherapy | 130 (48.5) |
| Hormone treatment | 72 (26.9) |
| Immunotherapy | 11 (4.1) |
| Bone marrow transplant | 8 (3.0) |
| Other treatment | 37 (13.8) |
| Current treatment ^h^ | 133 (49.6) |

Note. ^a^ 1 missing; ^b^ 2 missing; ^c^ low= primary and lower secondary education, intermediate = upper secondary education, high = higher vocational training/university; ^d^ 83 missing; ^e^ percentages do not add up to 100 because 26 patients had multiple types of cancer; ^f^ 4 missing; ^g^ percentages do not add up to 100 because patients followed multiple treatments; ^h^ 34 missing.

**Online Supplementary Table 7.2.** Labels and mean scores of the selected nodes in the partner

sample.

| Node (range) | M (SD) |
| --- | --- |
| Fatigue (7-56) | 36.20 (12.43) |
| Depressive mood (0-12) | 3.18 (2.47) |
| Loss of Enjoyment (4-24) | 12.73 (4.22) |
| Anxiety (0-3) | 1.08 (0.95) |
| Sleep problems (0-3) | 1.44 (1.01) |
| Concentration problems (0-3) | 1.40 (0.91) |
| Worthlessness (0-3) | 0.54 (0.82) |
| Appetite loss (0-3) | 0.37 (0.67) |
| Physical symptoms (0-36) | 13.84 (3.73) |
| Social withdrawal (8-40) | 20.60 (7.56) |
| Helplessness (6-24) | 12.82 (3.77) |
| Protective buffering by partner (8-40) | 19.80 (4.09) |
| Overprotection by partner (6-30) | 11.71 (3.67) |
| Acceptance of illness (6-24) | 12.65 (3.62) |
| Perceived benefits of illness (6-24) | 13.86 (4.24) |
| Disengagement of unattainable goals (4-20) | 10.77 (2.98) |
| Reengagement of new goals (6-30) | 20.54 (3.72) |
| Active engagement by partner (5-25) | 19.22 (4.03) |

**Online Supplementary Figure 7.1.** The network structure of symptoms and risk and protective factors of 268 cancer patients in the partner sample.


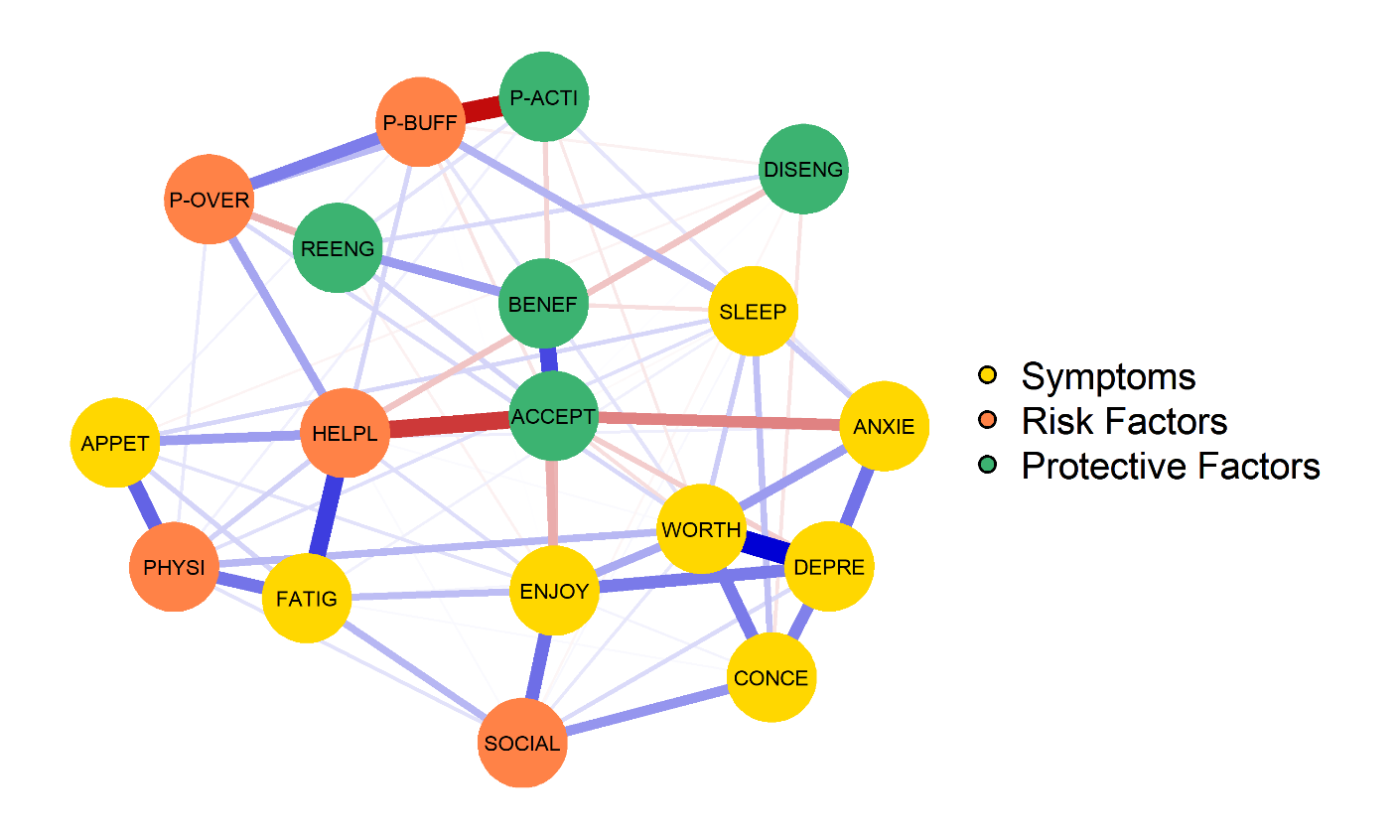


Note. The stronger a connection between two nodes, the thicker and more saturated the edge. Positive and negative connections are denoted by blue and red edges, respectively. FATIG = fatigue, DEPRE = depressed mood, ENJOY = loss of enjoyment, ANXIE = anxiety; SLEEP = sleep problemes, CONCE = concentration problems, WORTH = worthlessness, APPET = appetite loss, PHYSI = physical symptoms, SOCIAL = social withdrawal, HELPL = helplessness, P-BUFF = protective buffering by partner, P-OVER = overprotection by partner, ACCEPT = acceptance of illness, BENEF = perceived benefits of illness, DISENG = disengagement of unattainable goals; REENG = reengagement of new goals, P-ACTI = active engagement by partner.

Findings: Network of partner sample (n=268) appears similar to network of main sample (n=342). Edge weight difference test demonstrated that the following six edges were stronger than most other edges: depressed mood – worthlessness, acceptance – helplessness, acceptance – perceived benefits, fatigue – helplessness, physical symptoms – appetite loss, protective buffering – active engagement by partner. With the exception of the connection between protective buffering and active engagement these strong edges were also found in the main sample. The main sample also revealed the edges between depressed mood and loss of enjoyment and between fatigue and physical symptoms among the strongest edges.

**Online Supplementary Figure 7.2.** Strength centrality of each node in the network in the partner

sample.


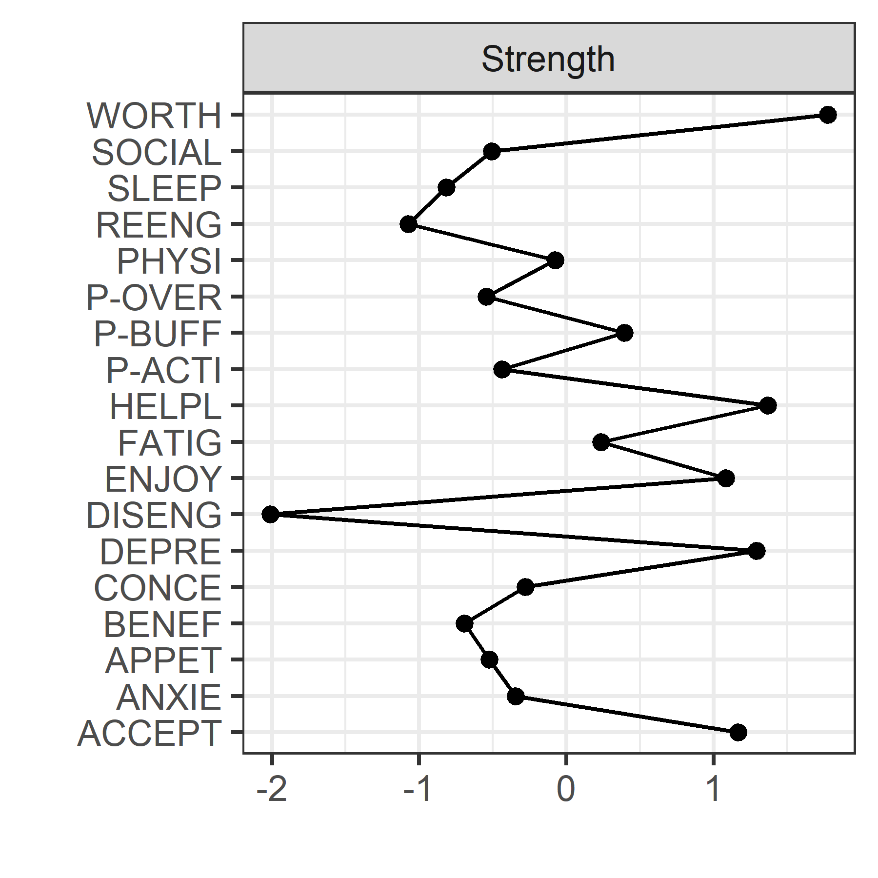


Findings. Node strength difference test demonstrated that in the partner sample (n=268) worthlessness, helplessness, depressed mood, acceptance and loss of enjoyment were the most central nodes in the network. With the exception of helplessness, these findings are similar to the centrality results in the main sample (n=342).
